# Evaluation of genetic variants related to lipid levels among the North Indian population

**DOI:** 10.3389/fgene.2023.1234693

**Published:** 2024-01-29

**Authors:** Gagandeep Kaur Walia, Jeemon Panniyammakal, Tripti Agarwal, Ruchita Jalal, Ruby Gupta, Lakshmy Ramakrishnan, Nikhil Tandon, Ambuj Roy, Anand Krishnan, Dorairaj Prabhakaran

**Affiliations:** ^1^ Public Health Foundation of India, New Delhi, India; ^2^ Centre for Chronic Disease Control, New Delhi, India; ^3^ Sree Chitra Tirunal Institute for Medical Sciences and Technology, Trivandrum, India; ^4^ Indian Institute of Public Health-Delhi, New Delhi, India; ^5^ All India Institute of Medical Science, New Delhi, India

**Keywords:** lipids, India, genetic variants, polygenic risk scores, weighted genetic risk scores

## Abstract

**Background:** A heavy burden of cardiometabolic conditions on low- and middle-income countries like India that are rapidly undergoing urbanization remains unaddressed. Indians are known to have high levels of triglycerides and low levels of HDL-C along with moderately higher levels of LDL-C. The genome-wide findings from Western populations need to be validated in an Indian context for a better understanding of the underlying etiology of dyslipidemia in India.

**Objective:** We aim to validate 12 genetic variants associated with lipid levels among rural and urban Indian populations and derive unweighted and weighted genetic risk scores (uGRS and wGRS) for lipid levels among the Indian population.

**Methods:** Assuming an additive model of inheritance, linear regression models adjusted for all the possible covariates were run to examine the association between 12 genetic variants and total cholesterol, triglycerides, HDL-C, LDL-C, and VLDL-C among 2,117 rural and urban Indian participants. The combined effect of validated loci was estimated by allelic risk scores, unweighted and weighted by their effect sizes.

**Results:** The wGRS for triglycerides and VLDL-C was derived based on five associated variants (rs174546 at *FADS1*, rs17482753 at *LPL*, rs2293889 at *TRPS1*, rs4148005 at *ABCA8*, and rs4420638 at *APOC1*), which was associated with 36.31 mg/dL of elevated triglyceride and VLDL-C levels (*β* = 0.95, SE = 0.16, *p* < 0.001). Similarly, every unit of combined risk score (rs2293889 at *TRPS1* and rs4147536 at *ADH1B*) was associated with 40.62 mg/dL of higher total cholesterol (*β* = 1.01, SE = 0.23, *p* < 0.001) and 33.97 mg/dL of higher LDL-C (*β* = 1.03, SE = 0.19, *p* < 0.001) based on its wGRS (rs2293889 at *TRPS1*, rs4147536 at *ADH1B*, rs4420638 at *APOC1*, and rs660240 at *CELSR2*). The wGRS derived from five associated variants (rs174546 at *FADS1*, rs17482753 at *LPL*, rs4148005 at *ABCA8*, rs4420638 at *APOC1*, and rs7832643 at *PLEC*) was associated with 10.64 mg/dL of lower HDL-C (*β* = −0.87, SE = 0.14, *p* < 0.001).

**Conclusion:** We confirm the role of eight genome-wide association study (GWAS) loci related to different lipid levels in the Indian population and demonstrate the combined effect of variants for lipid traits among Indians by deriving the polygenic risk scores. Similar studies among different populations are required to validate the GWAS loci and effect modification of these loci by lifestyle and environmental factors related to urbanization.

## Introduction

Lipid levels are established risk factors for cardiovascular disorders (Castelli et al., 1992; Benjamin et al., 2018) and are among the leading causes of morbidity and mortality (Mohebi et al., 2022). Globally, high cholesterol is estimated to cause 2.6 million deaths (4.5% of total deaths) and 29.7 million disability-adjusted life years (2.0% of total DALYs) (Noubiap et al., 2015). Dyslipidemia in the form of increased low-density lipoprotein cholesterol (LDL-C) levels, elevated triglyceride (TG) levels, and lower high-density lipoprotein cholesterol (HDL-C) levels is widely prevalent in India, with estimates ranging from 39% to 63% (Gupta et al., 2017).

Family and twin studies suggest familial aggregation of lipid disorders and relatively high heritability of various lipid traits. The heritability varies from ∼35% to 48% for TG, ∼40%–50% for LDL-C, and ∼40%–60% for HDL-C (Weiss et al., 2006). Furthermore, a family history of hyperlipidemia/dyslipidemia is associated with a 2.5–7-fold increase in the risk of death due to premature coronary artery disease compared to individuals without such family history (Sanghera et al., 2019). Genome-wide scans and their meta-analysis on different populations identified several loci associated with the serum lipid levels. Although more than 400 loci (Teslovich et al., 2010; Klarin et al., 2018; de Vries et al., 2019; Graff et al., 2021) have been identified cumulatively, these loci explain a small proportion of variances in blood lipid levels (Pilia et al., 2006). The therapeutic potential of some of the identified genome-wide association study (GWAS) signals in the management of dyslipidemia is widely explored (Klarin et al., 2018).

Most of the GWAS loci were replicated in the LOLIPOP cohort of Indian-origin people residing in the United Kingdom (Teslovich et al., 2010). However, considerable Asian/European differences in lipid profiles have been reported (Zhang et al., 2010). Few attempts have been made to validate the genome-wide findings in Indian populations previously (Braun et al., 2012; Rafiq et al., 2012; Arv et al., 2014; Walia et al., 2014; Pranavchand and Reddy, 2017). A new genome-wide scan among Indians living in India identified some novel loci that need further investigations for wider utility (Bandesh et al., 2019). In the present study, we aimed to validate some selected loci related to lipid levels based on their biological relevance in Indian rural and urban populations and derive polygenic risk scores for lipid levels among Indians.

## Methods

### Study population

The details about the original study that aimed to examine the 20-year trend of cardiometabolic risk factors in rural and urban areas around the National Capital Region are available in Prabhakaran et al. (2017). Urban participants were recruited from the Municipal Corporation of Delhi, and rural participants were recruited from Ballabgarh Block of the adjoining state of Haryana. For the present analyses, the follow-up blood samples were utilized for genotyping the selected variants related to lipid levels. Written informed consent was obtained from all the study participants to use their de-identified stored biological samples for future genetic research related to cardiometabolic risk factors. The present study was approved by the Institute Ethics Committee of the All India Institute of Medical Sciences, New Delhi and Centre for Chronic Disease Control, New Delhi.

### Phenotype data

Pre-informed written consent was obtained from each participant before beginning data collection. The details of the data collection have been described previously (Prabhakaran et al., 2017). Detailed questionnaires were administered to collect data on socio-demographic factors, medical history, and lifestyle factors, including diet, physical activity, tobacco smoking, and alcohol consumption. Daily fat consumption (g/day) was estimated from the detailed food frequency questionnaire, and the physical activity scores were categorized. The physical measures included anthropometry for height, weight, and waist and hip girths, along with blood pressure measurements. Fasting blood samples were collected from the participants, and the time of the last meal was recorded. Serum and plasma samples were used for generating data on glycemic and lipid levels. Plasma glucose was measured using the hexokinase method, total cholesterol was estimated using the enzymatic cholesterol oxidase method, and HDL-C was assayed by a direct method using PEG-modified enzymes and dextran sulfate using kits from Roche Diagnostics, Switzerland, on the c311 autoanalyzer (Roche). LDL-C was estimated using the Friedewald equation (Friedewald et al., 1972).

### Genotyping and quality control

The buffy coat samples were processed for the phenol–chloroform method for DNA isolation. The isolated DNA was then processed for genotyping of established GWAS variants using the multiplex Sequenom MassARRAY technology. These variants were selected based on their biological importance and high GWAS significance levels related to lipid traits. A detailed description of 12 genetic variants that were found in the Hardy–Weinberg equilibrium (HWE) is listed in Table 2. The HWE was estimated separately in rural and urban samples and also in the combined samples at a Sidak-corrected significance value (*p* < 0.0018). Power estimates were derived using Quanto software. The present study with high-quality genotype data (≥95% call rates) of 2,117 individuals had >80% power to estimate a 1% variation in lipid measures with a minimum risk allele frequency of 10% and *α* = 0.05. We did not apply for multiple testing corrections as the presently studied variants are established GWAS loci, and we aim to validate these loci in our Indian population.

### Statistical analysis

All analyses were performed using Stata v13.1. Depending on the skewness of the distribution, the continuous variables were suitably transformed and reported as mean (SD), whereas the categorical variables were summarized as number (%). The continuous variables were standardized (z-transformed) prior to any association analyses to facilitate comparability between coefficients.

Linear regression models were run for examining the association of each genetic variant with each of the lipid traits while assuming an additive model of inheritance. The regression model was fully adjusted for the related lifestyle factors, including fat intake, physical activity, BMI, alcohol consumption, smoking, hypertension, and diabetes. To estimate the combined effect of validated loci on each lipid level, allelic risk scores were calculated using loci significantly associated in both the regression models with respective lipid traits examined in the present study. We generated both weighted and unweighted allelic risk scores and compared the effect sizes of the two scores for each of the lipid traits. Weighted genetic risk scores (wGRS) weighing upon the internally derived effect sizes were calculated for each of the lipid traits using the following equation:

$$WeightedGeneticRiskScore=\left(\left(w1\times a1\right)+\ldots .+\left(wi\times ai\right)\right)$$

where w is the weight (effect size), a is the number of risk alleles, i is the total number of genetic variants, and N is the total number of risk alleles.

Since lipid levels are strongly associated with gender and urbanization due to underlying lifestyle factors, stratified analyses were also undertaken for gender and rural/urban sites while adjusting for all the confounders, as mentioned previously. We also examined the effect modification by gender, rural/urban sites, BMI, diet (fat intake), and physical activity by performing interaction analyses.

## Results

The characteristics of 2,117 participants are described in Table 1. All the variables except triglycerides and VLDL-C were normally distributed. Hence, the two variables were log-transformed for all subsequent analyses. The mean age of the participants was 47.1 (12.99) years. Less than half of the study participants (42.3%) were males. Male participants had significantly higher levels of triglycerides, VLDL-C, fasting plasma glucose, and blood pressure. On the other hand, female participants had higher HDL-C levels, BMI, and self-reported hypertension. Male participants also reported higher levels of physical activity, alcohol consumption, and smoking than female participants (Table 1). The rural participants had significantly higher levels of total cholesterol, HDL-C, LDL-C, average fat consumption, and smoking, whereas urban participants had higher BMI, fasting plasma glucose, systolic blood pressure, physical activity, alcohol consumption, and self-reported diabetes and hypertension (Table 1). The details of 12 genetic variants examined to be associated with lipid levels in the present study are listed in Table 2 along with their chromosomal location, nature of variation, and the functional implications of these variations. There were only three variants located in intergenic regions, namely, rs17482753 at *LPL*, rs2814944 at *C6orf106*, and rs4420638 at *APOC1*. Furthermore, only four of them were located in intronic regions, namely, rs2293889 at *TRPS1*, rs4148005 at *ABCA8*, rs4147536 at *ADH1B*, and rs7832643 at *PLEC*, and there were three 3′-UTR variants, namely, rs10773003 at *SBNO1*, rs174546 at *FADS1*, and rs660240 at *CELSR2* (Table 2). There were only two coding sequence variants, rs1800961 at *HNF4A* (a missense variant) and rs737337 at *DOCK6* (a synonymous variant) (Table 2). Of 12 examined variants, eight were observed to be significantly associated with one or more lipid traits in our study population (Table 3), and the absolute standardized effect sizes (*β*-coefficients from linear regression) ranged from 0.08 to 0.14.

**TABLE 1:** Characteristics of the study participants.

Characteristic	Total (N = 2,117)	Male (N = 958)	Female (N = 1,159)	*p*-value^1^	Urban (N = 958)	Rural (N = 1,159)	*p*-value^2^
Age (years) ^a^	47.07 (12.99)	47.99 (12.94)	46.32 (12.99)	0.032^c^	46.68 (13.02)	47.61 (12.94)	0.104
Gender^b^							0.254
Male	958 (45.25)	-	-	-	565 (26.69)	393 (18.56)	
Female	1,159 (54.75)	-	-	-	655 (30.94)	504 (23.81)	
Location^b^				0.254			
Urban	1,220 (57.63)	565 (26.69)	655 (30.94)		-	-	-
Rural	897 (42.37)	393 (18.56)	504 (23.81)		-	-	-
Total cholesterol (mg/dL) ^a^	183.68 (40.35)	183.53 (41.90)	183.79 (39.03)	0.884	181.05 (40.04)	187.37 (40.50)	<0.001^c^
Triglycerides^a^(mg/dL)^d^	125.96 (1.60)	136.01 (1.63)	118.23 (1.55)	<0.001^c^	126.89 (1.62)	124.68 (1.57)	0.399
HDL cholesterol (mg/dL) ^a^	46.97 (12.26)	44.41 (12.59)	49.07 (11.57)	<0.001^c^	46.04 (11.65)	48.27 (12.97)	<0.001^c^
LDL cholesterol (mg/dL) ^a^	108.10 (33.13)	107.72 (33.72)	108.42 (32.65)	0.630	105.92 (32.56)	111.16 (33.69)	<0.001^c^
VLDL cholesterol ^a^ (mg/dL)^d^	25.19 (1.60)	27.20 (1.63)	23.65 (1.55)	<0.001^c^	25.38 (1.62)	24.94 (1.57)	0.399
BMI (kg/m^2^) ^a^	24.91 (5.25)	24.16 (4.63)	25.53 (5.64)	<0.001^c^	26.28 (5.34)	23.06 (4.50)	<0.001^c^
Fasting plasma glucose (mg/dL) ^a^	108.83 (38.49)	111.32 (42.66)	106.77 (34.57)	0.007^c^	112.82 (42.28)	103.22 (31.62)	<0.001^c^
Systolic blood pressure (mm Hg) ^a^	127.79 (1.63)	130.76 (20.42)	125.34 (22.29)	<0.001^c^	130.14 (22.04)	124.62 (20.65)	<0.001^c^
Diastolic blood pressure (mm Hg) ^a^	82.83 (11.99)	84.60 (12.26)	81.38 (11.56)	<0.001^c^	83.13 (12.02)	82.43 (11.94)	0.182
Average daily fat intake (g/day) ^a^	31.09 (5.05)	29.92 (5.15)	32.06 (4.75)	<0.001^c^	30.19 (5.44)	32.30 (4.17)	<0.001^c^
Total physical activity/day^b^				<0.001^c^			<0.001^c^
Inactive	219 (11.50)	91 (10.10)	128 (12.76)		91 (7.80)	128 (17.34)	
Low active	1,004 (52.73)	488 (54.16)	516 (51.45)		624 (53.52)	380 (51.49)	
Moderately active	481 (25.26)	190 (21.09)	291 (29.01)		329 (28.22)	152 (20.60)	
Highly active	200 (10.50)	132 (14.65)	68 (6.78)		122 (10.46)	78 (10.57)	
Diabetes (self-reported)^b^	169 (7.98)	85 (4.02)	84 (3.98)	0.170	147 (6.94)	11 (0.52)	<0.001^c^
Hypertension (self-reported)^b^	400 (18.90)	152 (7.18)	248 (11.72)	0.001^c^	302 (14.27)	98 (4.63)	<0.001^c^
Alcohol consumption (yes)^b^	506 (23.90)	498 (23.52)	8 (0.38)	<0.001^c^	267 (12.61)	239 (11.29)	0.011^c^
Smoking (yes)^b^	498 (23.52)	377 (17.81)	121 (5.72)	<0.001^c^	143 (2.03)	355 (16.77)	<0.001^c^

^a^ All continuous variables are reported as mean (SD).

^b^ Categorical variables are reported as n (%).

^c^ Test of comparison (*t*-test and χ2 test depending on the variable) between groups; *p* < 0.05 signifies the groups are different for the variable.

^d^ Geometric mean.

**TABLE 2:** Description of the studied genetic variants related to lipid levels.

SNV	*Loci*	Chromosomal location	Change in nucleotide	Functional consequence	Minor allele	Minor allele frequency				
						**Combined**	**Rural**	**Urban**	**GWAS**	**MAF-GIH**
rs10773003	*SBNO1*	12:123290580	G→A	3′-UTR variant	A	0.11	0.10	0.12	0.16	0.10
rs174546	*FADS1*	11:61802358	C→T	3′-UTR variant	T	0.14	0.12	0.15	0.27	0.10
rs17482753	*LPL*	8:19975135	G→T	NA	T	0.08	0.08	0.07	0.11	0.09
rs1800961	*HNF4A*	20:44413724	C→T	Coding sequence variant missense amino acid change: T (Thr) > I (Ile)	T	0.02	0.02	0.02	0.03	0.03
rs2293889	*TRPS1*	8:115586972	G→T	Intronic variant	T	0.34	0.31	0.36	0.41	0.31
rs2814944	*C6orf106*	6:34585020	G→A	NA	A	0.10	0.11	0.10	0.16	0.11
rs4147536	*ADH1B*	4:99317955	G→T	Intronic variant	T	0.22	0.20	0.24	-----	0.30
rs4148005	*ABCA8*	17:68886325	A→C	Intronic variant downstream transcript variant	C	0.26	0.23	0.28	0.59	0.31
rs4420638	*APOC1*	19:44919689	A→G	Downstream transcript variant	G	0.11	0.10	0.11	0.16	-----
rs660240	*CELSR2*	1:109275216	G→A	3′-UTR variant	A	0.24	0.21	0.21	0.21	0.25
rs737337	*DOCK6*	19:11236817	T→C	Coding sequence variant synonymous amino acid change: (Thr) > T (Thr)	C	0.13	0.13	0.13	0.08	0.16
rs7832643	*PLEC*	8: 143948489	G→T	Intronic variant upstream transcript variant	T	0.30	0.27	0.32	0.40	0.34

SNV, single-nucleotide variation.

Change in nucleotide and minor allele information is based on the study population.

Information related to functional consequences reported from the NCBI-dbSNP database; NA, no detailed functional consequence was available in the dbSNP database.

**TABLE 3:** Association of genetic variants with lipid levels in the present study.

		Triglyceride			Total cholesterol			HDL-C			LDL-C			VLDL-C		
		*β*	*SE*	*p*-*value*	*β*	*SE*	*p*-*value*	*β*	*SE*	*p*-*value*	*β*	*SE*	*p*-*value*	*β*	*SE*	*p*-*value*
rs10773003	Model 1	−0.04	0.05	0.383	−0.02	0.05	0.721	0.00	0.05	0.990	−0.02	0.05	0.742	−0.04	0.05	0.383
	Model 2	−0.05	0.05	0.312	0.00	0.05	0.921	0.01	0.05	0.796	−0.01	0.05	0.909	−0.05	0.05	0.312
rs174546	Model 1	0.13	0.04	0.004^*^	−0.03	0.04	0.471	−0.11	0.04	0.010^*^	−0.06	0.04	0.181	0.13	0.04	0.004^*^
	Model 2	0.11	0.04	0.014^*^	−0.06	0.05	0.191	−0.10	0.04	0.025^*^	−0.09	0.04	0.036^*^	0.11	0.04	0.014^*^
rs17482753^$^	Model 1	0.19	0.06	0.001^*^	0.05	0.06	0.418	−0.13	0.06	0.018^*^	0.02	0.06	0.756	0.19	0.06	0.001^*^
	Model 2	0.21	0.06	<0.001^*^	0.06	0.06	0.332	−0.14	0.06	0.018^*^	0.03	0.06	0.662	0.21	0.06	<0.001^*^
rs1800961	Model 1	−0.05	0.11	0.668	−0.23	0.11	0.038^*^	−0.23	0.11	0.040^*^	−0.15	0.11	0.176	−0.05	0.11	0.668
	Model 2	−0.06	0.11	0.585	−0.21	0.12	0.066	−0.22	0.11	0.055	−0.13	0.12	0.260	−0.06	0.11	0.585
rs2293889	Model 1	0.08	0.03	0.023^*^	0.08	0.03	0.013^*^	−0.06	0.03	0.094	0.09	0.03	0.005^*^	0.08	0.03	0.023^*^
	Model 2	0.08	0.03	0.015^*^	0.10	0.03	0.004^*^	−0.06	0.03	0.062	0.11	0.03	0.001^*^	0.08	0.03	0.015^*^
rs2814944	Model 1	0.05	0.05	0.370	−0.03	0.05	0.583	0.07	0.05	0.152	−0.02	0.05	0.633	0.05	0.05	0.370
	Model 2	0.06	0.05	0.233	0.00	0.05	0.937	0.04	0.05	0.440	−0.06	0.05	0.235	0.06	0.05	0.233
rs4147536^$^	Model 1	0.04	0.04	0.259	0.10	0.04	0.004^*^	0.06	0.04	0.092	0.10	0.04	0.007^*^	0.04	0.04	0.259
	Model 2	0.05	0.04	0.187	0.12	0.04	0.001^*^	0.09	0.04	0.014^*^	0.10	0.04	0.006^*^	0.05	0.04	0.187
rs4148005	Model 1	0.09	0.03	0.010^*^	−0.04	0.03	0.299	−0.13	0.03	<0.001^*^	−0.05	0.03	0.187	0.09	0.03	0.010^*^
	Model 2	0.11	0.04	0.001^*^	−0.01	0.04	0.814	−0.13	0.03	<0.001^*^	−0.02	0.04	0.482	0.11	0.04	0.001^*^
rs4420638	Model 1	0.11	0.05	0.027^*^	0.08	0.05	0.123	−0.17	0.05	<0.001^*^	0.13	0.05	0.009^*^	0.11	0.05	0.027^*^
	Model 2	0.10	0.05	0.044^*^	0.07	0.05	0.191	−0.15	0.05	0.003^*^	0.11	0.05	0.028^*^	0.10	0.05	0.044^*^
rs660240^$^	Model 1	−0.01	0.04	0.734	0.09	0.04	0.013^*^	−0.01	0.04	0.734	0.12	0.04	0.001^*^	−0.01	0.04	0.734
	Model 2	0.00	0.04	0.933	0.07	0.04	0.059	−0.01	0.04	0.830	0.08	0.04	0.020^*^	0.00	0.04	0.933
rs737337	Model 1	0.05	0.04	0.300	−0.04	0.04	0.417	−0.08	0.04	0.068	−0.04	0.04	0.410	0.05	0.04	0.300
	Model 2	0.01	0.05	0.767	−0.03	0.05	0.533	−0.06	0.05	0.188	−0.02	0.05	0.716	0.01	0.05	0.767
rs7832643	Model 1	−0.02	0.03	0.600	−0.04	0.03	0.174	−0.11	0.03	0.001^*^	−0.01	0.03	0.761	−0.02	0.03	0.600
	Model 2	−0.02	0.03	0.507	−0.04	0.03	0.279	−0.09	0.03	0.004^*^	−0.01	0.03	0.879	−0.02	0.03	0.507
GRS	Model 1	0.10	0.02	<0.001^*^	0.09	0.02	<0.001^*^	−0.11	0.01	<0.001^*^	0.11	0.02	<0.001^*^	0.10	0.02	<0.001^*^
wGRS	Model 1	0.95	0.16	<0.001^*^	1.01	0.23	<0.001^*^	−0.87	0.14	<0.001^*^	1.03	0.19	<0.001^*^	0.95	0.16	<0.001^*^

Model 1—adjusted for age, gender, and site (urban/rural).

Model 2—adjusted for age, gender, site (urban/rural), fat intake, physical activity (inactive/low active/moderately active/highly active), BMI (kg/m^2^, alcohol consumption, smoking, hypertension, and diabetes.

*—level of significance = 0.05.

$—risk allele is the major allele.

GRS and wGRS (unweighted and weighted genetic risk score; generated using variants significantly associated in both models)—TG (rs174546, rs17482753^$^, rs2293889, rs4148005, and rs4420638), TC (rs2293889 and rs4147536^$^), HDL-C (rs174546, rs17482753^$^, rs4148005, rs4420638, and rs7832643); LDL-C (rs2293889, rs4147536^$^, rs4420638, and rs660240^$^), and VLDL-C (rs174546, rs17482753^$^, rs2293889, rs4148005, and rs4420638).

Each copy of the minor allele of rs174546 at *FADS1* was associated with a 1.05 mg/dL increase in triglyceride and VLDL-C levels (*β* = 0.11, SE = 0.04, *p* = 0.014), 1.21 mg/dL decrease in HDL-C levels (*β* = −0.10, SE = 0.04, *p* = 0.025), and 3.11 mg/dL decrease in LDL-C levels (*β* = −0.09, SE = 0.04, *p* = 0.036). The minor allele of rs17482753 at *LPL* was associated with an increase of 1.10 mg/dL in triglyceride and VLDL-C levels (*β* = 0.21, SE = 0.06, *p* < 0.001) and a reduction of 1.68 mg/dL in HDL-C levels (*β* = −0.14, SE = 0.06, *p* = 0.018). The rs2293889 at *TRPS1* was associated with a 1.04 mg/dL increase in triglyceride and VLDL-C levels (*β* = 0.08, SE = 0.03, *p* = 0.015), 4.06 mg/dL increase in total cholesterol (*β* = 0.10, SE = 0.03, *p* = 0.004), and 3.76 mg/dL increase in LDL-C levels (*β* = 0.11, SE = 0.01, *p* = 0.001). The rs4148005 at *ABCA8* was associated with a 1.06 mg/dL increase in triglycerides and VLDL-C levels (*β* = 0.11, SE = 0.04, *p* = 0.001) and 1.64 mg/dL decrease in HDL-C levels (*β* = −0.13, SE = 0.03, *p* < 0.001). The minor allele of rs4420638 at *APOC1* was associated with 1.05 mg/dL of elevated triglyceride and VLDL-C levels (*β* = 0.10, SE = 0.05, *p* = 0.044), 3.71 mg/dL of elevated LDL-C levels (*β* = 0.11, SE = 0.05, *p* = 0.028), and 1.85 mg/dL of reduced HDL-C levels (*β* = −0.15, SE = 0.05, *p* = 0.003). The rs4147536 at *ADH1B* variant was associated with a 4.88 mg/dL increase in total cholesterol (*β* = 0.12, SE = 0.04, *p* = 0.001), 3.44 mg/dL increase in LDL-C levels (*β* = 0.10, SE = 0.04, *p* = 0.006), and 1.11 mg/dL increase in HDL-C levels (*β* = 0.09, SE = 0.04, *p* = 0.014). In addition, rs660240 at *CELSR2* was associated with a 2.78 mg/dL increase in LDL-C levels (*β* = 0.08, SE = 0.04, *p* = 0.020). Furthermore, rs7832643 at *PLEC* (*β* = −0.09, SE = 0.03, *p* = 0.004) was associated with a 1.16 mg/dL decrease in HDL-C levels (Table 2).

The combined effect of all the variants validated for lipid levels was than the individual effect of the variants (Table 3). Every unit increase in the wGRS was associated with 36.31 mg/dL of elevated triglyceride and VLDL-C levels (*β* = 0.95, SE = 0.16, *p* < 0.001); 40.62 mg/dL of higher total cholesterol (*β* = 1.01, SE = 0.23, *p* < 0.001); 33.97 mg/dL of higher LDL-C levels (*β* = 1.03, SE = 0.19, *p* < 0.001); and 10.64 mg/dL of lower HDL-C levels (*β* = −0.87, SE = 0.14, *p* < 0.001). The effect sizes of the unweighted allelic risk scores were comparatively much lower than the weighted scores (Table 3).

For sensitivity analyses, we examined the effect of the genetic variants stratified by gender and rural/urban location, where some of the variants showed a significant association only in specific groups (Supplementary Tables S1, S2). Among the eight variants validated for various lipid traits in the present study, none showed interaction with the examined lifestyle factors for the respective lipid traits (Supplementary Table S3). However, some of these validated variants showed significant interaction associations for other lipid traits. For instance, rs174546 showed a significant interaction effect with gender for total cholesterol (*β* = 0.18, SE = 0.09, *p* = 0.046) and rs7832643 with obesity for total cholesterol (*β* = 0.10, SE = 0.03, *p* = 0.002) and LDL-C (*β* = 0.07, SE = 0.03, *p* = 0.030). On the other hand, rs4148005 showed a significant interaction effect with physical activity for total cholesterol (*β* = 0.10, SE = 0.04, *p* = 0.022) and LDL-C (*β* = 0.10, SE = 0.04, *p* = 0.026) and rs660240 for HDL-C (*β* = −0.09, SE = 0.04, *p* = 0.033) levels. In addition, rs10773003 at *SBNO1* showed a significant interaction with gender (*β* = −0.29, SE = 0.10, *p* = 0.003), site (*β* = −0.24, SE = 0.10, *p* = 0.018), and obesity (*β* = −0.14, SE = 0.05, *p* = 0.002) for HDL-C and with fat intake for total cholesterol (*β* = 0.24, SE = 0.10, *p* = 0.018), triglyceride, and VLDL-C levels (*β* = 0.23, SE = 0.10, *p* = 0.027). Similarly, rs2814944 at *C6orf106* showed a significant interaction with gender for LDL-C (*β* = 0.23, SE = 0.11, *p* = 0.032) and with physical activity for total cholesterol (*β* = 0.15, SE = 0.07, *p* = 0.029). Finally, rs1800961 at *HNF4A* (*β* = 0.51, SE = 0.23, *p* = 0.029) and rs737337 at *DOCK6* (*β* = −0.24, SE = 0.09, *p* = 0.009) were found to be interacting with gender for triglycerides and VLDL-C levels.

## Discussion

The overall goal of the present study was to validate the previously reported GWAS loci in rural and urban populations in Delhi, India. It is important to validate GWAS loci in different populations to determine their clinical relevance for drug therapies and preventing dyslipidemia and coronary artery disease. In this study, we validated eight variants in Indian populations that are involved in pathways of lipid transportation and metabolism, fat accumulation, alcohol metabolism, and transcription suppression. We could validate all four intronic variants and two of the three examined variants in the 3′-UTR region. The weighted allelic scores derived from these validated variants were associated with 36.31 mg/dL of elevated triglyceride and VLDL-C levels; 40.62 mg/dL of higher total cholesterol; 33.97 mg/dL of higher LDL-C levels; and 10.64 mg/dL of lower HDL-C levels. We observed that although the regression models of both unweighted and weighted allelic risk scores had comparable r^2^ and *p*-values, the effect sizes of the weighted scores were larger than those of the unweighted scores.

*ABCA8* encodes for a membrane-associated protein which is a member of the superfamily of ATP-binding cassette (ABC) transporters. ABC proteins transport various molecules across extra- and intracellular membranes. The encoded protein also regulates lipid metabolism as it is involved in the cholesterol efflux pathway. rs4148005 at *ABCA8* is an intronic downstream transcript variant that was found to be associated with triglyceride, VLDL-C, and HDL-C levels in the present study*. ADH1B* encodes for alcohol dehydrogenase 1B enzyme that metabolizes a wide variety of substrates, including ethanol, retinol, other aliphatic alcohols, hydroxysteroids, and lipid peroxidation products. It plays a major role in ethanol catabolism and is thus known to be strongly associated with lipid levels. rs4147536 at *ADH1B* is also an intronic variant and was found to be associated with total cholesterol and LDL-C levels in the present analysis. *TRPS1* (transcriptional repressor GATA binding 1) gene provides instructions for the synthesis of a protein that regulates the activity of many other genes. It encodes for a transcription factor protein and is known to be associated with dyslipidemia. An intronic variant rs2293889 at *TRPS1* was found to be associated with all the atherogenic lipid levels (total cholesterol, triglycerides, LDL-C, and VLDL-C) in the present study. *PLEC* encodes for plectin protein that is produced in different tissues like skin and muscles and is capable of interlinking different elements of the cytoskeleton. Recently, its role in the regulation of lipid levels has been shown. An intronic upstream transcript, rs7832643 at *PLEC*, was found to be associated with only HDL-C levels in the present study.

*CELSR2* encodes for cadherin EGF LAG seven-pass G-type receptor 2 protein, which is a part of the cadherin superfamily. These proteins are receptors involved in contact-mediated communication. The deficiency of CELSR2 is known to suppress lipid accumulation in hepatocytes and thus affect lipid levels. rs660240 at *CELSR2* is a 3′-UTR variant that was found to be associated with only LDL-C levels in the present study. *FADS1* encodes for fatty acid desaturase enzyme which regulates the unsaturation of fatty acids. It is known to play a role in hepatic lipid composition and fat accumulation and is thus associated with lipid levels in blood. rs174546 at *FADS1* is another 3′-UTR variant in the present study that was found to be associated with triglyceride, VLDL-C, and HDL-C levels.

*APOC1* resides within the apolipoprotein gene cluster and encodes for protein, which is a member of the apolipoprotein C1 family. It plays a major role in HDL-C and VLDL-C metabolisms and is also known to inhibit cholesteryl ester transfer protein (CETP). A downstream transcript variant, rs4420638 at *APOC1*, was found to be associated with all the lipid levels in the present study, except total cholesterol. *LPL* encodes for lipoprotein lipase enzyme that plays a critical role in breaking down fat into triglycerides, which are carried by lipoproteins from various organs to blood. It hydrolyzes triglyceride-rich particles and affects HDL-C levels in the blood. In accordance with this biological function, rs17482753 at *LPL* was found to be associated with triglyceride, VLDL-C, and HDL-C levels, although the specific functional consequence of this genetic variation is not well documented.

Some of these variants (*CELSR2*, *CETP*, and *LPL*) emerged as lead signals in a genome-wide study based on the Indian population for serum lipid levels (Bandesh et al., 2019), thus suggesting the universality of GWAS findings. They also reported few novel loci (QKI, REEP3, TMCC2, FAM129C, FAM241B, and LOC100506207) at a suggestive genome-wide significance level (Bandesh et al., 2019) that need to be examined in a larger set of samples in different populations of India. Previous validation studies in Indian populations have also confirmed the role of these genetic variants related to lipid levels. A large rural–urban sibling-pair study from India examined the role of few GWAS loci related to lipid levels and reported the role of *APOA1*, *APOA5*, *APOB*, *CETP*, *GCKR*, *LPL*, *LIPC*, *TRIB1*, and *CELSR2*–*PSRC1*–*SORT1* loci (Rafiq et al., 2012; Walia et al., 2014). They also reported effect modification of lipid variants with urban location and gender in the Indian population (Walia et al., 2014). The variants in the *CELSR2-PSRC1-SORT1* gene cluster were also observed to be associated with cholesterol and LDL-C levels among an Asian Indian cohort in Arv et al. (2014). Similarly, variants in the 11223.3 chromosomal region involving *APOA1*, *APOA4*, *APOA5*, and *APOC* loci were reported to be strongly influencing the lipid levels and dyslipidemia in studies from northern (Braun et al., 2012) and southern India (Pranavchand and Reddy, 2017).

It is well established that gender is strongly associated with lipid levels due to differences in associated lifestyle factors and metabolism, and thus, gender-specific thresholds have been advised for clinical implications across the globe. In addition, regional differences in dyslipidemia have been reported by large multi-centric studies with considerable differences among northern and southern Indian populations (Ebrahim et al., 2010; Joshi et al., 2014). The presently examined population belongs to the northern part of India, where people are known to consume a high-fat diet that affects the circulating lipid levels. Moreover, northern India experiences extreme winter seasons that are not present in other parts of India, which is again associated with the consumption of a high-fat diet in winter and festive seasons. Furthermore, India is currently undergoing urbanization, which in turn is associated with cardiometabolic disease burden, including dyslipidemia (Ebrahim et al., 2010; Gupta et al., 2017), and previous studies have demonstrated the effect modification of genetic variants of lipid traits with the urban location (Walia et al., 2014). Therefore, we also explored the interaction of the studied variants with gender, urban location, and related lifestyle factors to examine the effect modification of the validation loci. However, we did not have enough sample size to capture moderate effect modification effects. Nevertheless, we noticed few significant associations with adverse lifestyle factors that need to be replicated in studies with a larger sample size.

In conclusion, we confirm the role of eight previously reported GWAS loci in the Indian population, as well as loci that are involved in different lipid regulation pathways. The weighted allelic risk scores were found to be associated with high levels of lipids. The validated variants and allelic risk scores are useful in further analyses where these risk scores can be used as instruments for examining causal relationships. The effect modification noticed for urban location and related lifestyle factors needs to be investigated further in a larger set of samples for a more meaningful interpretation.

## Data Availability

The original contributions presented in the study are publicly available. This data can be found here: https://ftp.ncbi.nlm.nih.gov/dbgap/studies/phs003469/phs003469.v1.p1, https://www.ncbi.nlm.nih.gov/projects/gap/cgi-bin/study.cgi?study_id=phs003469.v1.p1, https://www.ncbi.nlm.nih.gov/gap/sstr/report/phs003469.v1.p1.

## Supplementary material

SUPPLEMENTARY TABLE S1 Association analysis of the genetic variants stratified for gender.

SUPPLEMENTARY TABLE S2 Association analysis of the genetic variants stratified for site (rural/urban).

SUPPLEMENTARY TABLE S3 Results of Gene*Environment interaction analysis.
