# Supplementary material for: Evaluation of genetic variants related to lipid levels among the North Indian population
Source: Front Genet. 2024 Jan 29;14:1234693. doi: 10.3389/fgene.2023.1234693 (PMC10859749; doi:10.3389/fgene.2023.1234693)
Supplement: Supplementary file 1 [file Table1.DOCX]

**Supplementary Tables**

**Table S1: Association analysis of the genetic variants stratified for gender**

|  |  | Triglycerides | | | Total Cholesterol | | | HDL-C | | | LDL-C | | | VLDL-C | | |
| --- | --- | --- | --- | --- | --- | --- | --- | --- | --- | --- | --- | --- | --- | --- | --- | --- |
|  |  | ***β*** | ***SE*** | ***p-value*** | ***β*** | ***SE*** | ***p-value*** | ***Β*** | ***SE*** | ***p-value*** | ***β*** | ***SE*** | ***p-value*** | ***β*** | ***SE*** | ***p-value*** |
| rs10773003 | **Male** | 0.02 | 0.07 | 0.755 | 0.09 | 0.07 | 0.241 | -0.13 | 0.07 | 0.063 | 0.11 | 0.07 | 0.131 | 0.02 | 0.07 | 0.755 |
|  | **Female** | 0.10 | 0.07 | 0.121 | 0.09 | 0.07 | 0.155 | -0.16 | 0.07 | 0.015* | 0.11 | 0.07 | 0.109 | 0.10 | 0.07 | 0.121 |
| rs174546 | **Male** | 0.10 | 0.07 | 0.145 | 0.01 | 0.07 | 0.846 | -0.04 | 0.07 | 0.584 | 0.04 | 0.07 | 0.552 | 0.10 | 0.07 | 0.145 |
|  | **Female** | 0.09 | 0.06 | 0.130 | 0.13 | 0.06 | 0.025* | -0.14 | 0.06 | 0.013* | 0.14 | 0.06 | 0.020* | 0.09 | 0.06 | 0.130 |
| rs17482753 | **Male** | 0.19 | 0.09 | 0.035* | 0.04 | 0.09 | 0.630 | -0.09 | 0.09 | 0.309 | 0.02 | 0.09 | 0.830 | 0.19 | 0.09 | 0.035* |
|  | **Female** | 0.23 | 0.08 | 0.003* | 0.09 | 0.08 | 0.262 | -0.17 | 0.08 | 0.028* | 0.08 | 0.08 | 0.328 | 0.23 | 0.08 | 0.003* |
| rs1800961 | **Male** | 0.19 | 0.18 | 0.296 | 0.27 | 0.18 | 0.143 | -0.37 | 0.18 | 0.035* | 0.21 | 0.18 | 0.240 | 0.19 | 0.18 | 0.296 |
|  | **Female** | 0.25 | 0.14 | 0.075 | 0.17 | 0.14 | 0.247 | -0.09 | 0.15 | 0.528 | 0.07 | 0.15 | 0.638 | 0.25 | 0.14 | 0.075 |
| rs2293889 | **Male** | 0.03 | 0.05 | 0.547 | 0.10 | 0.05 | 0.074 | -0.05 | 0.05 | 0.330 | 0.13 | 0.05 | 0.015* | 0.03 | 0.05 | 0.547 |
|  | **Female** | 0.11 | 0.04 | 0.011* | 0.09 | 0.04 | 0.031* | -0.06 | 0.04 | 0.138 | 0.09 | 0.04 | 0.040* | 0.11 | 0.04 | 0.011* |
| rs2814944 | **Male** | 0.05 | 0.08 | 0.550 | 0.07 | 0.08 | 0.352 | -0.05 | 0.08 | 0.551 | 0.17 | 0.08 | 0.027* | 0.05 | 0.08 | 0.550 |
|  | **Female** | 0.05 | 0.07 | 0.455 | 0.06 | 0.07 | 0.396 | -0.04 | 0.07 | 0.573 | 0.05 | 0.07 | 0.507 | 0.05 | 0.07 | 0.455 |
| rs4147536 | **Male** | 0.08 | 0.06 | 0.139 | 0.16 | 0.06 | 0.006* | -0.07 | 0.06 | 0.192 | 0.15 | 0.06 | 0.008* | 0.08 | 0.06 | 0.139 |
|  | **Female** | 0.00 | 0.05 | 0.976 | 0.08 | 0.05 | 0.095 | -0.10 | 0.05 | 0.044* | 0.06 | 0.05 | 0.194 | 0.00 | 0.05 | 0.976 |
| rs4148005 | **Male** | 0.15 | 0.05 | 0.007* | 0.04 | 0.05 | 0.428 | -0.11 | 0.05 | 0.041* | 0.01 | 0.05 | 0.912 | 0.15 | 0.05 | 0.007* |
|  | **Female** | 0.08 | 0.05 | 0.066 | 0.05 | 0.05 | 0.256 | -0.15 | 0.05 | 0.001* | 0.05 | 0.05 | 0.303 | 0.08 | 0.05 | 0.066 |
| rs4420638 | **Male** | 0.14 | 0.08 | 0.091 | 0.13 | 0.08 | 0.116 | -0.08 | 0.08 | 0.302 | 0.14 | 0.08 | 0.079 | 0.14 | 0.08 | 0.091 |
|  | **Female** | 0.09 | 0.06 | 0.151 | 0.04 | 0.06 | 0.558 | -0.20 | 0.06 | 0.001* | 0.11 | 0.06 | 0.095 | 0.09 | 0.06 | 0.151 |
| rs660240 | **Male** | 0.04 | 0.05 | 0.452 | 0.10 | 0.06 | 0.073 | -0.04 | 0.05 | 0.404 | 0.11 | 0.05 | 0.039* | 0.04 | 0.05 | 0.452 |
|  | **Female** | 0.04 | 0.05 | 0.451 | 0.04 | 0.05 | 0.347 | -0.02 | 0.05 | 0.652 | 0.07 | 0.05 | 0.170 | 0.04 | 0.05 | 0.451 |
| rs737337 | **Male** | 0.12 | 0.07 | 0.092 | 0.01 | 0.07 | 0.934 | -0.06 | 0.07 | 0.378 | 0.03 | 0.07 | 0.674 | 0.12 | 0.07 | 0.092 |
|  | **Female** | 0.09 | 0.06 | 0.127 | 0.07 | 0.06 | 0.243 | -0.06 | 0.06 | 0.371 | 0.02 | 0.06 | 0.795 | 0.09 | 0.06 | 0.127 |
| rs7832643 | **Male** | 0.01 | 0.05 | 0.854 | 0.08 | 0.05 | 0.104 | -0.14 | 0.05 | 0.007* | 0.05 | 0.05 | 0.317 | 0.01 | 0.05 | 0.854 |
|  | **Female** | 0.03 | 0.04 | 0.517 | 0.02 | 0.04 | 0.657 | -0.05 | 0.04 | 0.259 | 0.05 | 0.05 | 0.268 | 0.03 | 0.04 | 0.517 |
| *Model adjusted for age, site(urban/rural), fat intake, physical activity (inactive/low active/mod active/ high active) and BMI (kg/m2)*  *Level of significance = 0.05* | | | | | | | | | | | | | | | | |

**Table S2: Association analysis of the genetic variants stratified for site (rural/urban)**

|  |  | Triglycerides | | | Total Cholesterol | | | HDL-C | | | LDL-C | | | VLDL-C | | |
| --- | --- | --- | --- | --- | --- | --- | --- | --- | --- | --- | --- | --- | --- | --- | --- | --- |
|  |  | ***Β*** | ***SE*** | ***p-value*** | ***β*** | ***SE*** | ***p-value*** | ***β*** | ***SE*** | ***p-value*** | ***β*** | ***SE*** | ***p-value*** | ***β*** | ***SE*** | ***p-value*** |
| rs10773003 | **Rural** | 0.08 | 0.08 | 0.315 | 0.09 | 0.08 | 0.255 | -0.15 | 0.09 | 0.093 | 0.01 | 0.08 | 0.948 | 0.08 | 0.08 | 0.315 |
|  | **Urban** | 0.03 | 0.06 | 0.683 | 0.05 | 0.06 | 0.441 | -0.11 | 0.06 | 0.055 | 0.01 | 0.06 | 0.895 | 0.03 | 0.06 | 0.683 |
| rs174546 | **Rural** | 0.12 | 0.07 | 0.096 | 0.12 | 0.08 | 0.113 | -0.06 | 0.08 | 0.458 | 0.20 | 0.08 | 0.011* | 0.12 | 0.07 | 0.096 |
|  | **Urban** | 0.09 | 0.06 | 0.114 | 0.02 | 0.06 | 0.706 | -0.12 | 0.05 | 0.022* | 0.03 | 0.06 | 0.588 | 0.09 | 0.06 | 0.114 |
| rs17482753 | **Rural** | 0.11 | 0.09 | 0.210 | 0.08 | 0.09 | 0.365 | -0.14 | 0.10 | 0.171 | 0.10 | 0.09 | 0.308 | 0.11 | 0.09 | 0.210 |
|  | **Urban** | 0.26 | 0.08 | 0.001* | 0.03 | 0.08 | 0.668 | -0.13 | 0.07 | 0.066 | 0.03 | 0.08 | 0.702 | 0.26 | 0.08 | 0.001* |
| rs1800961 | **Rural** | 0.19 | 0.21 | 0.382 | 0.01 | 0.23 | 0.961 | -0.30 | 0.24 | 0.211 | 0.06 | 0.23 | 0.808 | 0.19 | 0.21 | 0.382 |
|  | **Urban** | 0.15 | 0.14 | 0.275 | 0.30 | 0.13 | 0.028* | -0.17 | 0.12 | 0.177 | 0.21 | 0.13 | 0.125 | 0.15 | 0.14 | 0.275 |
| rs2293889 | **Rural** | 0.12 | 0.05 | 0.027* | 0.04 | 0.06 | 0.485 | -0.10 | 0.06 | 0.114 | 0.03 | 0.06 | 0.571 | 0.12 | 0.05 | 0.027* |
|  | **Urban** | 0.06 | 0.04 | 0.167 | 0.13 | 0.04 | 0.002* | -0.05 | 0.04 | 0.249 | 0.16 | 0.04 | <0.0018 | 0.06 | 0.04 | 0.167 |
| rs2814944 | **Rural** | 0.15 | 0.09 | 0.087 | 0.03 | 0.09 | 0.715 | -0.05 | 0.10 | 0.624 | 0.03 | 0.09 | 0.733 | 0.15 | 0.09 | 0.087 |
|  | **Urban** | 0.00 | 0.07 | 0.995 | 0.02 | 0.07 | 0.738 | -0.10 | 0.06 | 0.106 | 0.07 | 0.06 | 0.262 | 0.00 | 0.07 | 0.995 |
| rs4147536 | **Rural** | 0.00 | 0.06 | 0.953 | 0.16 | 0.06 | 0.015* | -0.08 | 0.07 | 0.220 | 0.17 | 0.06 | 0.008* | 0.00 | 0.06 | 0.953 |
|  | **Urban** | 0.06 | 0.05 | 0.185 | 0.10 | 0.05 | 0.039* | -0.10 | 0.04 | 0.027* | 0.07 | 0.05 | 0.149 | 0.06 | 0.05 | 0.185 |
| rs4148005 | **Rural** | 0.10 | 0.06 | 0.073 | 0.01 | 0.06 | 0.854 | -0.10 | 0.06 | 0.102 | 0.04 | 0.06 | 0.475 | 0.10 | 0.06 | 0.073 |
|  | **Urban** | 0.12 | 0.05 | 0.007* | 0.00 | 0.04 | 0.990 | -0.15 | 0.04 | <0.001* | 0.01 | 0.04 | 0.851 | 0.12 | 0.05 | 0.007* |
| rs4420638 | **Rural** | 0.06 | 0.08 | 0.442 | 0.01 | 0.08 | 0.862 | -0.14 | 0.09 | 0.108 | 0.02 | 0.08 | 0.780 | 0.06 | 0.08 | 0.442 |
|  | **Urban** | 0.12 | 0.07 | 0.062 | 0.11 | 0.06 | 0.075 | -0.16 | 0.06 | 0.008* | 0.17 | 0.06 | 0.009* | 0.12 | 0.07 | 0.062 |
| rs660240 | **Rural** | 0.04 | 0.06 | 0.512 | 0.02 | 0.06 | 0.805 | 0.00 | 0.07 | 0.994 | 0.00 | 0.06 | 0.991 | 0.04 | 0.06 | 0.512 |
|  | **Urban** | 0.03 | 0.05 | 0.584 | 0.12 | 0.04 | 0.007* | -0.01 | 0.04 | 0.783 | 0.14 | 0.04 | 0.002* | 0.03 | 0.05 | 0.584 |
| rs737337 | **Rural** | 0.01 | 0.07 | 0.936 | 0.01 | 0.07 | 0.942 | -0.01 | 0.08 | 0.909 | 0.01 | 0.07 | 0.886 | 0.01 | 0.07 | 0.936 |
|  | **Urban** | 0.02 | 0.06 | 0.798 | 0.05 | 0.06 | 0.371 | -0.09 | 0.05 | 0.099 | 0.05 | 0.06 | 0.434 | 0.02 | 0.06 | 0.798 |
| rs7832643 | **Rural** | 0.06 | 0.05 | 0.235 | 0.07 | 0.06 | 0.232 | -0.14 | 0.06 | 0.018* | 0.02 | 0.06 | 0.723 | 0.06 | 0.05 | 0.235 |
|  | **Urban** | 0.01 | 0.04 | 0.901 | 0.02 | 0.04 | 0.676 | -0.07 | 0.04 | 0.067 | 0.01 | 0.04 | 0.808 | 0.01 | 0.04 | 0.901 |
| *Model 2 adjusted for age, gender, fat intake, physical activity (inactive/low active/mod active/ high active) and BMI (kg/m2)*  *Level of significance = 0.05* | | | | | | | | | | | | | | | | |

**Table S3: Results of Gene*Environment interaction analysis**

|  | Triglycerides | | | | | | Total Cholesterol | | | HDL-C | | | LDL-C | | | VLDL-C | | |
| --- | --- | --- | --- | --- | --- | --- | --- | --- | --- | --- | --- | --- | --- | --- | --- | --- | --- | --- |
|  | ***β*** | | ***SE*** | | ***p-value*** | | ***β*** | ***SE*** | ***p-value*** | ***β*** | ***SE*** | ***p-value*** | ***β*** | ***SE*** | ***p-value*** | ***β*** | ***SE*** | ***p-value*** |
|  | 1. **Gene * Gender** | | | | | | | | | | | | | | | | | |
| rs10773003 | 0.17 | | 0.10 | | 0.094 | | 0.15 | 0.10 | 0.139 | -0.29 | 0.10 | 0.003* | 0.19 | 0.10 | 0.051 | 0.17 | 0.10 | 0.094 |
| rs174546 | 0.06 | | 0.09 | | 0.499 | | 0.18 | 0.09 | 0.046* | -0.10 | 0.09 | 0.246 | 0.13 | 0.09 | 0.159 | 0.06 | 0.09 | 0.499 |
| rs17482753 | 0.02 | | 0.12 | | 0.893 | | 0.03 | 0.12 | 0.818 | -0.08 | 0.12 | 0.507 | 0.08 | 0.12 | 0.477 | 0.02 | 0.12 | 0.893 |
| rs1800961 | 0.51 | | 0.23 | | 0.029* | | 0.07 | 0.23 | 0.765 | -0.29 | 0.23 | 0.199 | 0.12 | 0.23 | 0.604 | 0.51 | 0.23 | 0.029* |
| rs2293889 | 0.06 | | 0.07 | | 0.391 | | 0.01 | 0.07 | 0.941 | 0.00 | 0.07 | 0.985 | 0.03 | 0.07 | 0.612 | 0.06 | 0.07 | 0.391 |
| rs2814944 | 0.01 | | 0.11 | | 0.933 | | 0.14 | 0.11 | 0.183 | 0.00 | 0.10 | 0.988 | 0.23 | 0.11 | 0.032* | 0.01 | 0.11 | 0.933 |
| rs4147536 | 0.08 | | 0.07 | | 0.290 | | 0.09 | 0.08 | 0.210 | -0.02 | 0.07 | 0.797 | 0.10 | 0.07 | 0.165 | 0.08 | 0.07 | 0.290 |
| rs4148005 | 0.09 | | 0.07 | | 0.214 | | 0.11 | 0.07 | 0.135 | -0.04 | 0.07 | 0.547 | 0.06 | 0.07 | 0.388 | 0.09 | 0.07 | 0.214 |
| rs4420638 | 0.06 | | 0.10 | | 0.592 | | 0.10 | 0.10 | 0.338 | -0.12 | 0.10 | 0.246 | 0.04 | 0.10 | 0.693 | 0.06 | 0.10 | 0.592 |
| rs660240 | 0.06 | | 0.07 | | 0.427 | | 0.05 | 0.07 | 0.462 | -0.05 | 0.07 | 0.449 | 0.05 | 0.07 | 0.519 | 0.06 | 0.07 | 0.427 |
| rs737337 | -0.24 | | 0.09 | | 0.009* | | -0.11 | 0.11 | 0.223 | 0.00 | 0.09 | 0.984 | 0.02 | 0.09 | 0.857 | -0.24 | 0.09 | 0.009* |
| rs7832643 | -0.03 | | 0.07 | | 0.677 | | 0.11 | 0.07 | 0.119 | -0.09 | 0.07 | 0.154 | 0.11 | 0.07 | 0.122 | -0.03 | 0.07 | 0.677 |
|  | 1. **Gene * Site** | | | | | | | | | | | | | | | | | |
| rs10773003 | 0.07 | | 0.10 | | 0.526 | 0.15 | | 0.10 | 0.150 | -0.24 | 0.10 | 0.018* | 0.00 | 0.10 | 0.970 | 0.07 | 0.1 | 0.526 |
| rs174546 | 0.05 | | 0.10 | | 0.590 | 0.09 | | 0.10 | 0.354 | -0.06 | 0.09 | 0.518 | 0.16 | 0.10 | 0.092 | 0.05 | 0.1 | 0.590 |
| rs17482753 | 0.14 | | 0.12 | | 0.244 | 0.06 | | 0.12 | 0.621 | -0.01 | 0.12 | 0.939 | 0.13 | 0.12 | 0.282 | 0.14 | 0.1 | 0.244 |
| rs1800961 | 0.34 | | 0.26 | | 0.193 | 0.30 | | 0.26 | 0.249 | -0.14 | 0.26 | 0.582 | 0.25 | 0.26 | 0.332 | 0.34 | 0.3 | 0.193 |
| rs2293889 | 0.07 | | 0.07 | | 0.332 | 0.09 | | 0.07 | 0.239 | -0.05 | 0.07 | 0.438 | 0.12 | 0.07 | 0.096 | 0.07 | 0.1 | 0.332 |
| rs2814944 | 0.16 | | 0.11 | | 0.169 | #REF! | | 0.11 | 0.573 | -0.16 | 0.11 | 0.155 | 0.05 | 0.11 | 0.651 | 0.16 | 0.1 | 0.169 |
| rs4147536 | 0.05 | | 0.08 | | 0.562 | 0.07 | | 0.08 | 0.374 | -0.02 | 0.08 | 0.812 | 0.11 | 0.08 | 0.163 | 0.05 | 0.1 | 0.562 |
| rs4148005 | 0.02 | | 0.07 | | 0.764 | 0.02 | | 0.07 | 0.830 | -0.05 | 0.07 | 0.461 | 0.04 | 0.07 | 0.583 | 0.02 | 0.1 | 0.764 |
| rs4420638 | 0.03 | | 0.11 | | 0.765 | 0.12 | | 0.11 | 0.250 | -0.01 | 0.10 | 0.907 | 0.14 | 0.11 | 0.183 | 0.03 | 0.1 | 0.765 |
| rs660240 | 0.07 | | 0.08 | | 0.382 | 0.14 | | 0.08 | 0.066 | -0.01 | 0.08 | 0.907 | 0.14 | 0.08 | 0.064 | 0.07 | 0.1 | 0.382 |
| rs737337 | 0.01 | | 0.10 | | 0.891 | 0.07 | | 0.10 | 0.492 | -0.11 | 0.09 | 0.256 | 0.07 | 0.10 | 0.453 | 0.01 | 0.1 | 0.891 |
| rs7832643 | 0.07 | | 0.07 | | 0.324 | 0.05 | | 0.07 | 0.449 | -0.07 | 0.07 | 0.332 | 0.03 | 0.07 | 0.658 | 0.07 | 0.1 | 0.324 |
|  | 1. **Gene * Obesity** | | | | | | | | | | | | | | | | | |
| rs10773003 | 0.03 | 0.05 | | 0.504 | | 0.07 | | 0.05 | 0.162 | -0.14 | 0.05 | 0.002* | 0.07 | 0.05 | 0.125 | 0.03 | 0.05 | 0.504 |
| rs174546 | 0.02 | 0.04 | | 0.664 | | 0.01 | | 0.04 | 0.812 | -0.01 | 0.04 | 0.758 | 0.01 | 0.04 | 0.816 | 0.02 | 0.04 | 0.664 |
| rs17482753 | 0.00 | 0.05 | | 0.989 | | 0.00 | | 0.06 | 0.983 | -0.02 | 0.05 | 0.670 | 0.01 | 0.05 | 0.896 | 0.00 | 0.05 | 0.989 |
| rs1800961 | 0.11 | 0.11 | | 0.325 | | 0.02 | | 0.11 | 0.871 | -0.09 | 0.11 | 0.387 | 0.01 | 0.11 | 0.913 | 0.11 | 0.11 | 0.325 |
| rs2293889 | 0.06 | 0.03 | | 0.084 | | 0.01 | | 0.03 | 0.820 | -0.01 | 0.03 | 0.713 | 0.02 | 0.03 | 0.458 | 0.06 | 0.03 | 0.084 |
| rs2814944 | 0.04 | 0.05 | | 0.431 | | 0.02 | | 0.05 | 0.760 | -0.07 | 0.05 | 0.147 | 0.02 | 0.05 | 0.690 | 0.04 | 0.05 | 0.431 |
| rs4147536 | 0.02 | 0.04 | | 0.622 | | 0.03 | | 0.04 | 0.449 | 0.00 | 0.04 | 0.989 | 0.04 | 0.04 | 0.305 | 0.02 | 0.04 | 0.622 |
| rs4148005 | 0.00 | 0.03 | | 0.921 | | 0.01 | | 0.03 | 0.815 | 0.00 | 0.03 | 0.912 | 0.00 | 0.03 | 0.927 | 0.00 | 0.03 | 0.921 |
| rs4420638 | 0.01 | 0.05 | | 0.887 | | 0.05 | | 0.05 | 0.286 | -0.04 | 0.05 | 0.399 | 0.08 | 0.05 | 0.124 | 0.01 | 0.05 | 0.887 |
| rs660240 | 0.02 | 0.03 | | 0.574 | | 0.06 | | 0.04 | 0.087 | -0.06 | 0.03 | 0.070 | 0.04 | 0.03 | 0.307 | 0.02 | 0.03 | 0.574 |
| rs737337 | 0.02 | 0.05 | | 0.723 | | 0.00 | | 0.05 | 0.922 | -0.01 | 0.05 | 0.828 | 0.01 | 0.05 | 0.909 | 0.02 | 0.05 | 0.723 |
| rs7832643 | 0.05 | 0.03 | | 0.107 | | 0.10 | | 0.03 | 0.002* | -0.06 | 0.03 | 0.063 | 0.07 | 0.03 | 0.030* | 0.05 | 0.03 | 0.107 |
|  | 1. **Gene * Fat intake** | | | | | | | | | | | | | | | | | |
| rs10773003 | 0.23 | 0.10 | | 0.027* | | 0.24 | | 0.10 | 0.018* | -0.11 | 0.10 | 0.288 | 0.14 | 0.10 | 0.167 | 0.23 | 0.10 | 0.027* |
| rs174546 | 0.17 | 0.09 | | 0.061 | | 0.05 | | 0.09 | 0.577 | -0.04 | 0.09 | 0.642 | 0.14 | 0.09 | 0.115 | 0.17 | 0.09 | 0.061 |
| rs17482753 | 0.00 | 0.12 | | 0.982 | | 0.02 | | 0.12 | 0.869 | -0.05 | 0.12 | 0.657 | 0.09 | 0.12 | 0.468 | 0.00 | 0.12 | 0.982 |
| rs1800961 | 0.29 | 0.24 | | 0.211 | | 0.07 | | 0.24 | 0.766 | -0.30 | 0.23 | 0.197 | 0.04 | 0.23 | 0.850 | 0.29 | 0.24 | 0.211 |
| rs2293889 | 0.05 | 0.07 | | 0.445 | | 0.05 | | 0.07 | 0.438 | -0.01 | 0.07 | 0.927 | 0.09 | 0.07 | 0.192 | 0.05 | 0.07 | 0.445 |
| rs2814944 | 0.20 | 0.11 | | 0.066 | | 0.03 | | 0.11 | 0.753 | -0.19 | 0.11 | 0.067 | 0.02 | 0.11 | 0.879 | 0.20 | 0.11 | 0.066 |
| rs4147536 | 0.11 | 0.08 | | 0.139 | | 0.00 | | 0.08 | 0.954 | -0.07 | 0.07 | 0.324 | 0.00 | 0.08 | 0.983 | 0.11 | 0.08 | 0.139 |
| rs4148005 | 0.02 | 0.07 | | 0.728 | | 0.07 | | 0.07 | 0.309 | -0.10 | 0.07 | 0.153 | 0.01 | 0.07 | 0.874 | 0.02 | 0.07 | 0.728 |
| rs4420638 | 0.04 | 0.10 | | 0.702 | | 0.02 | | 0.10 | 0.821 | -0.12 | 0.10 | 0.245 | 0.05 | 0.10 | 0.629 | 0.04 | 0.10 | 0.702 |
| rs660240 | 0.04 | 0.07 | | 0.612 | | 0.03 | | 0.07 | 0.696 | 0.00 | 0.07 | 0.980 | 0.02 | 0.07 | 0.827 | 0.04 | 0.07 | 0.612 |
| rs737337 | 0.03 | 0.09 | | 0.788 | | 0.09 | | 0.10 | 0.331 | -0.04 | 0.09 | 0.701 | 0.12 | 0.09 | 0.190 | 0.03 | 0.09 | 0.788 |
| rs7832643 | 0.04 | 0.07 | | 0.538 | | 0.04 | | 0.07 | 0.536 | -0.03 | 0.07 | 0.666 | 0.00 | 0.07 | 0.973 | 0.04 | 0.07 | 0.538 |
|  | 1. **Gene * Physical Activity** | | | | | | | | | | | | | | | | | |
| rs10773003 | 0.05 | 0.06 | | 0.429 | | 0.01 | | 0.06 | 0.858 | -0.05 | 0.06 | 0.409 | 0.03 | 0.06 | 0.580 | 0.05 | 0.06 | 0.429 |
| rs174546 | 0.07 | 0.06 | | 0.186 | | 0.00 | | 0.06 | 0.941 | -0.05 | 0.06 | 0.416 | 0.01 | 0.06 | 0.821 | 0.07 | 0.06 | 0.186 |
| rs17482753 | 0.07 | 0.07 | | 0.307 | | 0.03 | | 0.07 | 0.663 | -0.03 | 0.07 | 0.617 | 0.06 | 0.07 | 0.405 | 0.07 | 0.07 | 0.307 |
| rs1800961 | 0.06 | 0.13 | | 0.675 | | 0.10 | | 0.14 | 0.475 | -0.13 | 0.13 | 0.321 | 0.05 | 0.13 | 0.721 | 0.06 | 0.13 | 0.675 |
| rs2293889 | 0.00 | 0.04 | | 0.989 | | 0.02 | | 0.04 | 0.625 | -0.07 | 0.04 | 0.086 | 0.02 | 0.04 | 0.713 | 0.00 | 0.04 | 0.989 |
| rs2814944 | 0.05 | 0.07 | | 0.449 | | 0.15 | | 0.07 | 0.029* | -0.03 | 0.07 | 0.606 | 0.13 | 0.07 | 0.062 | 0.05 | 0.07 | 0.449 |
| rs4147536 | 0.03 | 0.05 | | 0.550 | | 0.05 | | 0.05 | 0.280 | -0.07 | 0.05 | 0.110 | 0.05 | 0.05 | 0.307 | 0.03 | 0.05 | 0.550 |
| rs4148005 | 0.02 | 0.04 | | 0.690 | | 0.10 | | 0.04 | 0.022* | -0.07 | 0.04 | 0.123 | 0.10 | 0.04 | 0.026* | 0.02 | 0.04 | 0.690 |
| rs4420638 | 0.05 | 0.06 | | 0.454 | | 0.06 | | 0.06 | 0.377 | -0.03 | 0.06 | 0.594 | 0.08 | 0.06 | 0.242 | 0.05 | 0.06 | 0.454 |
| rs660240 | 0.01 | 0.04 | | 0.784 | | 0.03 | | 0.05 | 0.571 | -0.09 | 0.04 | 0.033* | 0.00 | 0.04 | 0.983 | 0.01 | 0.04 | 0.784 |
| rs737337 | 0.03 | 0.06 | | 0.576 | | 0.05 | | 0.06 | 0.364 | -0.10 | 0.05 | 0.078 | 0.01 | 0.06 | 0.807 | 0.03 | 0.06 | 0.576 |
| rs7832643 | 0.06 | 0.04 | | 0.148 | | 0.00 | | 0.04 | 0.992 | 0.00 | 0.04 | 0.905 | 0.01 | 0.04 | 0.825 | 0.06 | 0.04 | 0.148 |
| β refers to effect size from interaction term  Regression Model adjusted for:  (i) Age, gender, *site(urban/rural)*, fat intake, physical activity (inactive/low active/mod active/ high active), BMI (kg/m2) and *genotype variable*  (ii) Age, gender, *site(urban/rural)*, fat intake, physical activity (inactive/low active/mod active/ high active), BMI (kg/m2) and *genotype variable*  (iii) Age, gender, site(urban/rural), fat intake, physical activity (inactive/low active/mod active/ high active), *BMI(Categorical)* and *genotype variable*  (Iv) Age, gender, site(urban/rural), BMI (kg/m2), physical activity (inactive/low active/mod active/ high active), Fat intake (Categorical) and genotype variable  (v) Age, gender, site(urban/rural), BMI (kg/m2), Fat intake, physical activity (inactive/low active/mod active/ high active) and genotype variable  Level of significance = 0.05 | | | | | | | | | | | | | | | | | | |
